# Dysbiosis of small intestinal microbiota in liver cirrhosis and its association with etiology

**DOI:** 10.1038/srep34055

**Published:** 2016-09-30

**Authors:** Yanfei Chen, Feng Ji, Jing Guo, Ding Shi, Daiqiong Fang, Lanjuan Li

**Affiliations:** 1 State Key Laboratory for Diagnosis and Treatment of Infectious Disease, Collaborative Innovation Center for Diagnosis and Treatment of Infectious Diseases, The First Affiliated Hospital, Zhejiang University, Hangzhou 310003, PR China; 2 Department of Gastroenterology, The First Affiliated Hospital, Zhejiang University, Hangzhou 310003, PR China

## Abstract

Cirrhosis-associated duodenal dysbiosis is not yet clearly defined. In this research, duodenal mucosal microbiota was analyzed in 30 cirrhotic patients and 28 healthy controls using 16S rRNA gene pyrosequencing methods. The principal coordinate analysis revealed that cirrhotic patients were colonized by remarkable different duodenal mucosal microbiota in comparison with controls. At the genus level, *Veillonella*, *Megasphaera*, *Dialister*, *Atopobium*, and *Prevotella* were found overrepresented in cirrhotic duodenum. And the duodenal microbiota of healthy controls was enriched with *Neisseria*, *Haemophilus*, and *SR1 genera incertae sedis*. On the other hand, based on predicted metagenomes analyzed, gene pathways related to nutrient absorption (e.g. sugar and amino acid metabolism) were highly abundant in cirrhosis duodenal microbiota, and functional modules involved in bacterial proliferation and colonization (e.g. bacterial motility proteins and secretion system) were overrepresented in controls. When considering the etiology of cirrhosis, two operational taxonomic units (OTUs), OTU-23 (*Neisseria*) and OTU-36 (*Gemella*), were found discriminative between hepatitis-B-virus related cirrhosis and primary biliary cirrhosis. The results suggest that the structure of duodenal mucosa microbiota in cirrhotic patients is dramatically different from healthy controls. The duodenum dysbiosis might be related to alterations of oral microbiota and changes in duodenal micro-environment.

Gut microbiota and bacterial translocation (BT) play an important role in the pathogenesis of complications of cirrhosis^1^. Although a lot of work has been done to characterize the gut microbiota in cirrhosis, fecal samples were used in most of these studies. The small intestine has been suggested as the preferred site for BT in cirrhosis^2^. Small intestinal bacterial overgrowth (SIBO) has the greatest potential for promoting BT. This is supported by the study showing bacterial translocate predominantly from the small intestines when inoculating equal concentrations of *E.coli* into small or large intestines^3^. Moreover, reduction in BT rates is associated with lower jejunal, but not cecal, bacterial counts in cisapride-treated cirrhotic rats with an increased intestinal motility^4^. SIBO is traditionally defined if bacterial culture more than 10^5^ CFU/ml in upper jejuna aspirate. However, the majority of intestine microbiota could not be cultured, so the culture-based method cannot reveal the real changes of microbiota in the small intestines^6^.

Recently, the duodenal microbiota has been analyzed in a few cohort studies using 16S rRNA gene pyrosequencing method. Duodenal samples demonstrated greater biological diversity and possessed a unique microbial signature compared with the rectum^7^. The duodenal microbiota of obese individuals was found to have a higher proportion of anaerobic genera and a lesser proportion of aerobic genera in comparison with normal-weight individuals^8^. Altered duodenal microbiota composition was also demonstrated in celiac disease patients suffering from persistent symptoms on a long-term gluten-free diet^9^. The treated patients with persistent symptoms had reduced microbial richness with higher relative abundance of *Proteobacteria* and lower abundance of *Bacteroidetes* and *Firmicutes*. In 2011, Steed *et al*. analyzed the microbial composition of duodenal biopsies samples in cirrhosis by real-time PCR, but found no significant difference between controls and cirrhosis^10^. The results might be hampered by the small sample size, as only 6 distal duodenal biopsies were evaluated in their study.

We hypothesized that the alterations of upper gastrointestinal tract in cirrhosis would lead to dysbiosis of duodenal microbiota. The aim of this study was to characterize the duodenal mucosal microbiota of cirrhotic patients using 16S rRNA pyrosequencing methods. The results demonstrated that cirrhotic patients were colonized by remarkable different duodenal microbiota in comparison with healthy controls.

## Results

### Clinical characteristics and pyrosequencing data summary

A total of 58 subjects were included in this duodenal mucosal microbial profiling study. Characteristics of the 30 cirrhotic patients and 28 healthy controls, including demorgraphics, liver biochemistries, and pyrosequencing results are summarized in Table 1. Of the cirrhotics, 24 patients (80%) were Hepatitis-B-virus (HBV) related, 6 patients (20%) were primary biliary cirrhosis (PBC). Statistical comparisons of liver biochemistries revealed significantly higher (p < 0.05) levels of aminotransferase, aspartate aminotransferase, total bilirubin, alkaline phosphatase in cirrhotic group as compared to healthy controls. There were significant decline of blood hemoglobin and platelet count in cirrhosis group than in control group. Additional characteristics such as age, body mass index, and gender distribution were generally matched across the cirrhosis group and control group.

A total of 433,180 high-quality sequences were produced, accounting for 81.2% of valid sequences (average sequence length 464 bp), with an average of 7,225 reads per sample in control group and 7696 reads per sample in cirrhotic group. To normalize sequencing depth, subsets of 4,135 reads (the number of the sample with the least number of reads) per sample were picked randomly for alpha diversity comparison. No significant difference of community diversity and richness was observed between cirrhosis and controls.

### Compositional and functional analysis of duodenal microbiota in cirrhosis

**Figure 1:**
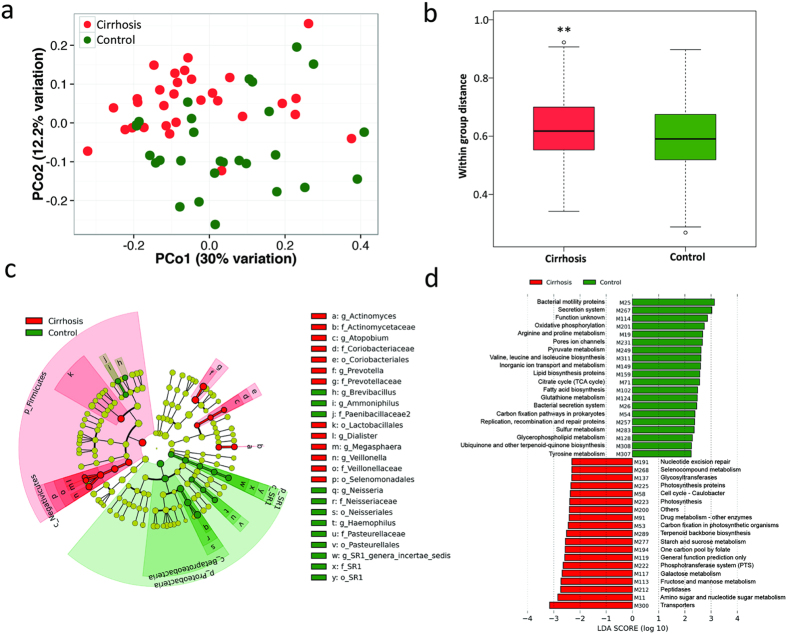
(**a**) Principal Coordinate Analysis of weighted UniFrac distance of 16S rRNA genes. The key at left indicated the types of subjects: cirrhosis (red), control (green). (**b**) Comparison of within group distance between cirrhosis group (red bar) and control group (green bar). **Indicate significant difference with p-value < 0.01. (**c**) Cladogram representing the taxonomic hierarchical structure of the identified phylotype biomarkers generated using LEfSe. Each filled circle represents one phylotype. Red, phylotypes statistically overrepresented in cirrhosis; green, phylotypes overrepresented in controls. Phylum and class are indicated in their names on the cladogram and the order, family, or genera are given in the key. (**d**) LEfSe based on the PICRUSt data set revealed differentially enriched bacterial functions associated either with cirrhosis (red) or controls (green). The Linear Discriminant Analysis (LDA) score at the log_10_ scale is indicated at the bottom. The greater the LDA score is, the more significant the functional biomarker is in the comparison.

Principal coordinate analysis based on weighted UniFrac matrices revealed remarkable differentiation of bacterial communities between cirrhosis and controls (Fig. 1a). When comparing the within-group variance, it was observed that the duodenal microbiota in cirrhosis have significantly higher variance than in controls (p < 0.01), which suggests that duodenal microbiota across cirrhosis patients are less similar than across healthy individuals (Fig. 1b).

To identify the distinguishing taxa in the duodenal microbiota of cirrhosis and controls, linear discriminant analysis effect size (LEfSe) were performed based on the RDP taxonomy data (Fig. 1c). At the phylum level, *Firmicutes* was significantly enriched in cirrhosis group. And *Proteobacteria* and *SR1* were more abundant in control group than in cirrhosis group. At the genus level, *Veillonella*, *Megasphaera*, *Dialister*, *Atopobium*, and *Prevotella* were found overrepresented in cirrhosis duodenum. And the duodenal microbiota of healthy controls was enriched with *Neisseria*, *Haemophilus*, and *SR1 genera incertae sedis*.

To investigate the functional potential of duodenal microbiota, phylogenetic investigation of communities by reconstruction of unobserved states (PICRUSt) analysis was applied to generate the prediction profiles of kyoto encyclopedia of genes and genomes modules. Functional modules were compared between cirrhosis and controls with LEfSe (Fig. 1d). The functional modules involved in bacterial motility proteins and secretion system were highly abundant in healthy control group. Additionally, modules for arginine and proline metabolism, valine, leucine and isoleucine biosynthesis were also enriched in control group. Cirrhosis was associated with increased abundance of modules involved in transporters and amino sugar and nucleotide sugar metabolism. Functional modules involved in peptidases and fructose and mannose metabolism were also overrepresented in cirrhosis group.

### Key OTUs in the duodenal microbiota of cirrhosis

**Figure 2:**
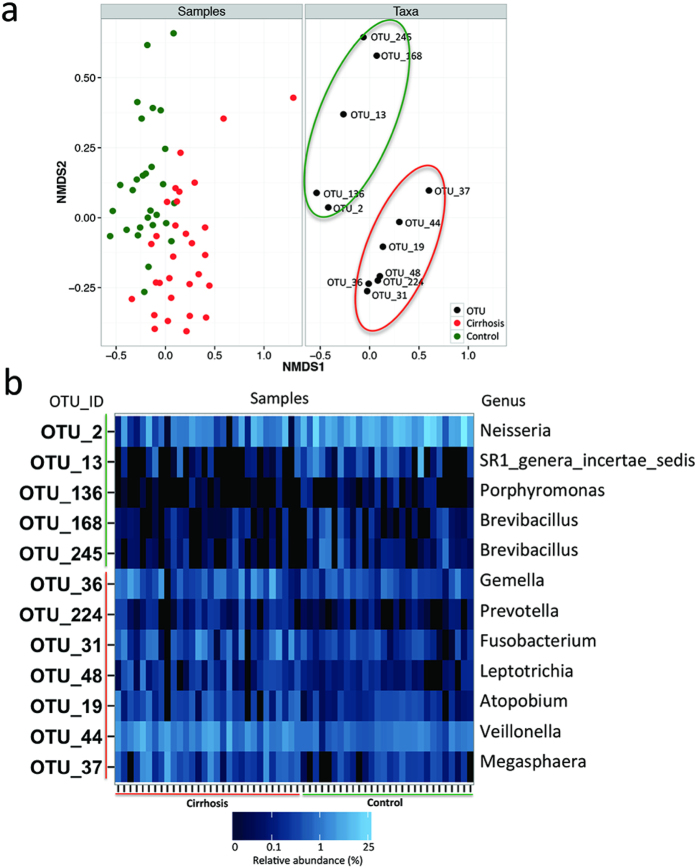
(**a**) Nonmetric multidimensional scaling biplot of duodenal samples based on 12 key OTUs identified by PLS-DA. On the left panel, each point represents one sample. The duodenal samples showed a clear separation between cirrhosis (red) and controls (green). On the right panel, each point represents one OTU. The OTUs in the red oval (7 OTUs) showed higher abundance in cirrhosis group. The OTUs in the green oval (5 OTUs) showed higher abundance in control group. (**b**) Heat map of relative abundance for the 12 key OTUs identified by PLS-DA. The relative abundance of each OTU in each subject of this study was used to plot the heat map. Each column represents one subject. The group information was showed under the plot: cirrhosis patients on the left with red line, controls on the right with black line. Each row represents one OTU. The genus affiliation of the OTU was showed on the right. The OTUs in red were found enriched in cirrhosis group, and in green enriched in controls.

Using partial least squares-discriminant analysis (PLS-DA), there were 12 OTUs identified as the key lineages contributing to the differentiation between cirrhosis and control duodenal microbiota. A clear separation between cirrhosis group and control group can be obtained by non-metric multidimensional scaling method based on these 12 key OTUs (Fig. 2a). Seven of these OTUs were found prevalent in cirrhosis group, along with other 5 OTUs declined in cirrhosis group (Fig. 2b). The OTU with the largest contrition to differentiation (OTU-2 with the variable importance in the projection value 2.3) belonged to *Neisseria* at the genus level.

### OTUs associated with etiology of cirrhosis

**Figure 3:**
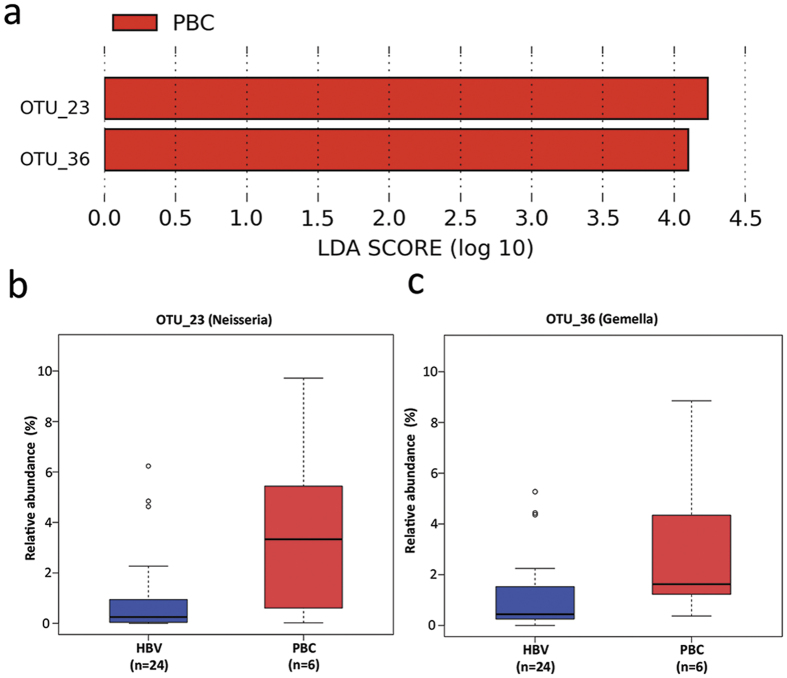
(**a**) Identified OTU biomarkers between cirrhosis of different etiologies. The graph was generated using the LEfSe program comparing samples from HBV-related cirrhosis and PBC at the OTU level. Both biomarker OTUs are overrepresented in PBC group. The LDA score at the log_10_ scale is indicated at the bottom. (**b**) Box plots of the relative abundance of biomarker OTUs in cirrhosis of different etiologies. On the left panel, OTU-23 (genus Neisseria) in HBV-related cirrhosis group and PBC group. On the right panel, OTU-36 (genus Gemella) in HBV-related cirrhosis group and PBC group. The body of the box plot represents the first and third quartiles of the distribution, and the median. The whiskers extend from the quartiles to the last data point within 1.5 *IQR, with outliers beyond represented as dots.

HBV-related cirrhosis and PBC were two main etiologies of cirrhosis in this study. When considering the etiology of cirrhosis, no significant difference was observed between HBV-related cirrhosis and PBC at the genus level or above. However, when LEfSe was applied on the OTU profile, there were two OTUs, OTU-23 (*Neisseria*) and OTU-36 (*Gemella*), discriminative between the two types of cirrhosis (Fig. 3a). Both of these two OTUs showed higher relative abundance in PBC than in HBV-related cirrhosis patients (Fig. 3b).

### Microbiota alterations and other clinical factors

**Figure 4:**
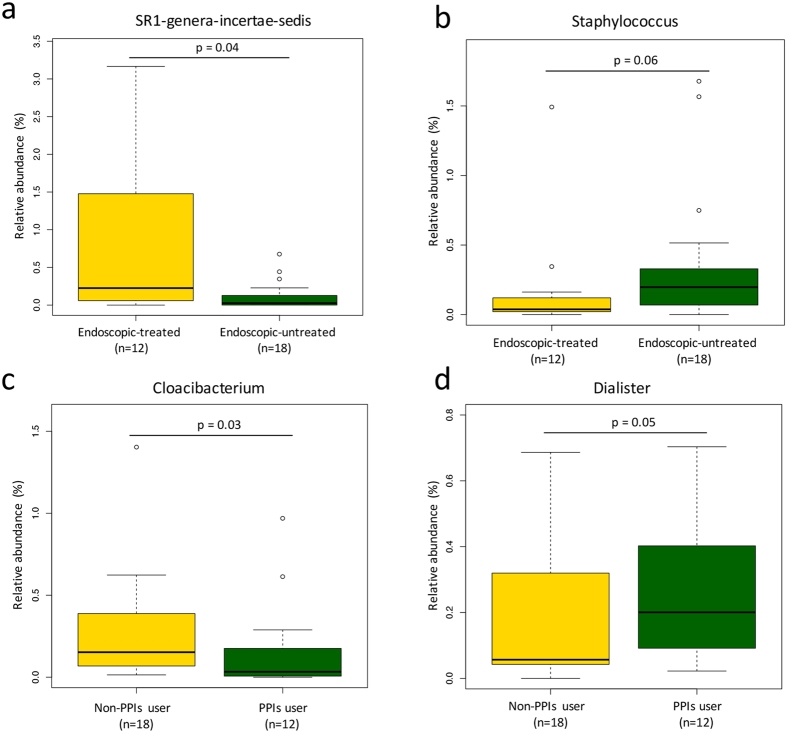
(**a**) Box plots of the relative abundance of genus SR1-genera-incertae-sedis between cirrhotic patients with endoscopic treatment and those without. (**b**) Box plots of the relative abundance of genus Staphylococcus between cirrhotic patients with endoscopic treatment and those without. (**c**) Box plots of the relative abundance of genus Cloacibacterium between cirrhotic patients on PPIs and those not. (**d**) Box plots of the relative abundance of genus Dialister between cirrhotic patients on PPIs and those not. Horizontal bars represent false discovery rate-corrected p value < 0.05 (Mann-Whitney U test). The body of the box plot represents the first and third quartiles of the distribution, and the median. The whiskers extend from the quartiles to the last data point within 1.5 *IQR, with outliers beyond represented as dots.

All the cirrhotic patients were detected with esophageal and gastric varices. Among of them, 12 cirrhotic patients had received endoscopic treatment for esophageal or gastric varices. The microbiota composition was compared between patients with endoscopic treatment (n = 12) versus those without (n = 18). Generally, the duodenal microbiota was similar at the family level or above. Endoscopic treatment was not associated with alterations of predicted functional genes. At the genus level, *SR1 genera incertae sedis* was found significantly higher in patients with endoscopic treatment than those without (median 0.23% in treated group versus 0.03% in untreated group, p = 0.04) (Fig. 4a). There was a moderate decrease of genus *Staphylococcus* in treated group than in untreated group (median 0.04% in treated group versus 0.20% in untreated group, p = 0.059) (Fig. 4b).

Microbiota composition between patients on proton pump inhibitors (PPIs) (n = 12) and those without (n = 18) was also compared. No significant difference was observed at the family level or above. PPIs did not change the profile of predicted functional genes. At the genus level, *Cloacibacterium* was significantly reduced in patients on PPIs (median 0.15% in patients on PPIs versus 0.03% in those without, p = 0.03) (Fig. 4c). Patients on PPIs were found to have moderate higher relative abundance of *Dialister* than those without (median 0.20% in patients on PPIs versus 0.06% in those without, p = 0.054) (Fig. 4d).

## Discussion

Studies of the duodenal microbiota have been focused on SIBO, which relies on traditional culture-dependent method or breath testing. Cirrhosis-associated duodenal dysbiosis is not yet clearly defined. In the attempt to shed light on this issue, we used 16S rRNA metagenomics to determine whether the duodenum microbiota differed between cirrhotic patients and healthy controls. Our results suggest that the structure of duodenal mucosa microbiota in cirrhotic patients is dramatically different from normal controls.

As can be observed in this study, at the genus level, *Veillonella*, *Prevotella, Neisseria,* and *Haemophilus*, were the most discriminative taxa between cirrhosis and controls. All these taxa are commonly presented in the oral cavity^11^, which suggests oral microbiota has a great impact upon duodenal microbiota. The oral cavity is the entry point of bacteria into the body. The human oral microbiota not only play a role in disease of the oral cavity, but also interact with microbiomes from other parts of the human body^12^. Our earlier study has found that microbes of oral origin could be present in stool of cirrhosis patients^13^. Recently, a direct evaluation of the salivary microbiome in controls and patients with cirrhosis was performed by Bajaj *et al*.^14^. In salivary microbiome of cirrhotic patients with previous hepatic encephalopathy, relative abundance of autochthonous taxa (*Lachnospiraceae* and *Ruminococcaceae*) decreased whereas potentially pathogenic taxa (*Prevotella* and *Fusobacteriaceae*) increased. Partly in consistent with their results, our results also found the relative abundance of duodenal *Prevotella* and *Fusobacterium* in cirrhosis was significantly higher than in controls. It has been reported in a previous study that distinct bacterial populations in the oral microbiota are involved in production of high levels of H_2_S and CH_3_SH in the oral cavity. The H_2_S group showed higher proportions of the genera *Neisseria*, *Porphyromonas* and *SR1*, whereas the CH_3_SH group had higher proportions of the genera *Prevotella*, *Veillonella*, *Atopobium*, *Megasphaera*, and *Selenomonas*^15^. It is interesting that the duodenal bacterial groups enriched in cirrhosis and healthy controls are highly consistent with the oral microbiota in CH_3_SH group and H_2_S group, respectively. Blood levels of CH_3_SH have been suggested as important factors in the pathogenesis of hepatic encephalopathy^16^. The shift of microbiota toward CH_3_SH generated community might indicate a direct contribution of duodenal microbiota to hepatic encephalopathy in cirrhosis. Our results are in line with recently published data showing that gut dysbiosis links with systemic and neuro-inflammation. When compared with control mice, the small intestinal microbiota in cirrhotic mice showed relative increase in *Enterobacteriaceae* and *Staphylococcaceae* along with predominantly oral families such as *Streptococcaceae*^17^. Taking together, these results might indicate associations between small intestinal microbiota of oral origins and hepatic encephalopathy.

To investigate if, in addition to compositional impact, there is an impact on bacterial function, PICRUSt was applied to the 16S rRNA amplicon sequencing data to infer bacterial metabolic functions. Interestingly, we found higher abundance of bacterial motility proteins and bacterial secretion system in healthy duodenal microbiota. The motility proteins play an important role in bacterial attachment on epithelial cells and travel to or away from stimulus^18^. Bacterial secretion system, which can be classified into type I-IV, operates generally on the principal of active transportation of protein from cytoplasm to bacterial surface, which play crucial roles in gut colonization through invasion on mucosal surface^19^. Both bacterial motility and secretion system are heavily involved in host adhesion and colonization. The enrichment of genes related to bacterial motility and secretion system might indicate a harsher and more competitive environment in healthy duodenum than in cirrhotic duodenum. In normal conditions, intestinal peristalsis, gastric acid, and bile secretion act to control control bacterial colonization, attachment, and infiltration into the host^20^. Abnormalities in one or more of these host defenses result in bacterial overgrowth of the small intestine^2^. In cirrhosis, marked decreases in intestinal intraluminal concentrations of bile acids have been ascribed to decreased secretion and increased deconjugation^21^. Abnormalities in small intestinal motility are related to the degree of chronic liver failure^22^. Decompensated cirrhotics were found to have slower intestinal transit times as compared with compensated cirrhotics and healthy controls^23^. Small intestine motility dysfunction is more severe in those with history of spontaneous bacterial peritonitis^24^. In our research, cirrhotic patients were observed with significantly higher inter-individual variations than healthy controls, which also supports the conclusion that cirrhosis abolish colonization control to certain exogenous bacteria and weaken the normal control of endogenous bacterial community.

The enriched pathways in cirrhosis were related to transporters, amino sugar and nucleotide sugar metabolism, which likely reflect the basic requirements of microbial life in the duodenum of cirrhosis. The bacterial transport systems enable bacteria to accumulate needed nutrients, extrude unwanted by products and maintain cytoplasmic content of protons and salts conducive to growth and development^25^. Some ABC transporter can be involved in resistance to antimicrobial peptides^26^. The drug transporters genes are overrepresented in the infant/elderly gut microbiome, perhaps due to the frequent antibiotic treatment of infants and the elderly compared with adults^27^. Therefore, we speculate that the enrichment of transporter gene might be a selective result of occasionally antibiotics use in cirrhotic patients, who are more susceptible to infections.

Although, at the genus level, *Neisseria* were found overrepresented in healthy controls. Two OTUs representing *Gemella* and *Neisseria*, respectively, were the most discriminative OTUs between two types of cirrhosis. The results here are in line with our recently published data, which showed alterations and correlations of the gut microbiome and immnunity in PBC patients^28^. The fecal microbiota of PBC patients were depleted of some potentially beneficial bacteria, such as *Lachnobacterium* and *Acidobacteria*, but were enriched in some bacterial taxa, such as *Neisseriaceae* and *Klebsiella*. Several altered gut bacterial taxa exhibited interactions with altered immunity and urine metabolism, such as *Klebsiella* with IL-2A and *Neisseriaceae* with urinary indoleacrylate. A close association between celiac disease and PBC has been extensively reported in literature^29^. In duodenum of adult celiac patients, members of *Neisseria* genus were significantly more abundant in active celiac disease patients than in controls^30^. *Gemella* has been found to be involved in pulmonary exacerbations of cystic fibrosis patients. The relative abundance of *Gemella* in airway microbiota increased in 83% of the patients with cystic fibrosis at exacerbation and was found to be the most discriminative genus between baseline and exacerbation samples^31^. It is hypothesized that specific species of *Neisseria* and *Gemella* may be involved in orchestrating inflammatory disease by promoting inflammation and remodeling normally benign microbiota into dysbiotic community. Recently, Sabino *et al*. found that primary sclerosing cholangitis is associated with alterations in intestinal microbiota independently of comorbidity with inflammatory bowel disease, which might indicate a potential effect of cholestatic and bile flow on gut microbiota^32^. Further studies are needed to confirm and assess these links and causality.

Our results showed that the duodenal microbiota is primary determined by cirrhosis itself. Only slight association can be observed between duodenal microbial alterations and endoscopy varices treatment or PPIs. An increase in the relative abundance of genus *Dialister* was observed in cirrhotic patients on PPIs. Certain *Dialister* species (*D. pnermosintes*, *D. invisus*) have been identified as pathogens, mainly in orthodontic infections^33^. It is hypothesized that PPIs could enhance gastrointestinal proliferation of these potential oral pathogen species by reduction in gastric acid. This hypothesis is supported by recent findings showing long-term PPIs use is associated with an increase of *Holdemania filiformis*^34^, which is also potential oral pathogens usually isolated from advanced periodontitis^35^. In the present study, genus *SR1 genera incertae sedis* and *Staphylococcus* were found increased and decreased in patients with endoscopic treatment, respectively. Endoscopic sclerotherapy and band ligation of esophageal varices have been showed to cause gastric hemodynamic changes, and thus increase the incidence and the severity of portal hypertensive gastropathy^36^. The duodenal microbiota alterations observed here might be related to mucosa changes in advanced portal hypertension. However, the present study was not designed to compare microbiota alterations before and after endoscopy treatment or PPIs use. Further large scale studies should be performed to confirm these links.

In conclusion, the study reported herein demonstrates that marked dysbiosis is associated with the duodenal mucosa of cirrhotic patients. The deviation of duodenum microbiota might be related to alterations of oral microbiota and duodenal microenvironment. Also, when considering the etiology of cirrhosis, PBC and HBV have slight difference of gut microbiota, indicating the effect mediated by immunity or bile acids between host and microbiota. We note that our study is only able to describe correlations between cirrhosis and duodenal microbiota, and causality cannot be inferred. Further studies, perhaps using experimental animals, are expected to shed light on the causal factors underlying this relationship.

## Methods

### Patients

The patients and controls recruitment and sampling were carried out at the first affiliated hospital of Zhejiang University. A total of 30 cirrhotic patients and 28 healthy subjects were recruited prior to attending for a pre-arranged upper gastrointestinal tract endoscopy in the hospital. All individuals underwent complete evaluation including biochemical tests of liver functions and ultrasongraphy. Cirrhosis was diagnosed on the basis of clinical and laboratory data supported by liver biopsy or ultrasongraphy. Patients were excluded if they were on or had received antibiotics in the last 2 months. Medication history in the last 2 months was recorded for each cirrhotic patient. There were 12 patients on PPIs. No other antacid medicine, rifaximin, or lactulose, was used in the cirrhotic patients. Child-Pugh score was used to assess the prognosis of cirrhosis^37^. Of the 30 patients included, there were 27 Child A, 2 Child B, and 1 Child C. None of the patients had a history of hepatic encephalopathy. All the cirrhotic patients were detected with esophageal and gastric varices. A total of 12 cirrhotic patients had received previous endoscopy varices sclerotherapy or ligation. One biopsy sample was taken from the distal duodenum of each individual. All participants provided written informed consent prior to entering the study. The study conformed to the ethical guidelines of the 1975 Declaration of Helsinki. The Institutional Review Board of the First Affiliated Hospital of Zhejiang University approved the study protocol on November 11^th^, 2014.

### DNA extraction from the mucosa

Total genomic DNA was extracted from the biopsy samples by using a combination of the QIAamp DNA isolation kit (Qiagen, Valencia, CA, USA) and a bead-beating method. Briefly, the biopsy sample was lysed in 180 μl of QIAamp ATL buffer and 20 μl of proteinase K for 1 h at 56 °C. Glass beads of different diameters (0.1 mm, 0.5 mm and 1 mm, Sigma, St. Louis, MO, USA) were added, and samples were homogenized in a FastPrep FP120 bead beater (Bio 101, Morgan Irvine, CA, USA) for 30 sec at 4 m/s and incubated for an additional hour at 56 °C. Four ml of RNase A (100 mg/ml) and 200 μl of AL buffer were added to the lysate, and samples were incubated for 30 min at 70 °C. After the addition of 200 μl absolute ethanol, lysates were purified over a QIAamp column as specified by the manufacturer. Samples were eluted in 200 μl of AE buffer.

### 16S rRNA gene pyrosequencing

PCR amplification of the bacterial 16S rRNA gene V1–V3 region was performed using universal primers (27F 5′-AGAGTTTGATCCTGGCTCAG-3′, 533R 5′-TTACCGCGGCTGCTGGCAC-3′). Details for PCR conditions, DNA purification were described previously^38^. Massive partial reads of 16S rRNA gene generated by the 454 GS FLX Titanium sequencer were initially trimmed for quality using standard the software tools from Roche/454.

### Bioinformatics and statistics

The 16S rRNA reads were processed and compared using QIIME pipeline 1.7.0^39^. The raw reads were trimmed using a minimum read length of 200 bp and an average quality score of 25. Two mismatches were allowed along the primer sequences. The number of hompolymers authorized in sequences was limited to 6. OTUs were picked using denovo OTU picking protocol with a 97% similarity threshold. The most abundant sequence of each OTU was selected as representative reads, and compared to the RDP classifier (cutoff = 0.5) taxonomy assignments of OTUs. The chimera identification sequence was performed by USEARCH^40^.

The QIIME results were imported into Phyloseq, an R package, for manipulation, subsampling normalization, and graph visualization^41^. The software PICRUSt was used to make functional gene content predictions based on 16S rRNA gene data present in the Greengenes database^42^. LEfSe was used to elucidate taxa and genes associated with healthy or diseased states. Both PICRUSt and LEfSe are freely available online in the Galaxy workflow framework^43^. To compare the alpha diversity and weighted unifrac distance between groups, the Two-sided student’s t-test was used for significance test. PLS-DA with the variable importance in the projection parameter was used to explore key OTUs contributing to differentiation between groups. Variable with the variable importance in the projection parameter >1.5 is defined as key OTUs associated with differentiation. Mann-Whitney U test was applied to compare genera abundances between different groups, with multiple testing correction whenever applicable (adjustment for false discovery rate). Adjusted p values <0.05 were considered significant. The t-test, non-parametric test, and PLS-DA projection were conducted in R (V.3.1.3) with package “mixOmics”, “plyr” and “reshape 2”.

## Additional Information

**How to cite this article**: Chen, Y. *et al*. Dysbiosis of small intestinal microbiota in liver cirrhosis and its association with etiology. *Sci. Rep.* **6**, 34055; doi: 10.1038/srep34055 (2016).

## Figures and Tables

**Table 1:** Clinical characteristics and pyrosequencing data summary.

	Healthy controls (n = 28)	Liver cirrhosis patients (n = 30)	p-value
*Demographics*			
Age (yr)	52 ± 9	49 ± 8	0.17
Gender (M/F)	22/6	23/7	0.86
BMI (kg/m^2^)	22.9 ± 2.8	22.8 ± 3.3	0.84
*Liver biochemistries*			
Etiology (HBV/PBC)	N/A	24/6	
Alb (g/L)	44.0 ± 1.8	36.5 ± 7.5	<0.001
TB (umol/L)	15.5 ± 3.0	21.9 ± 13.5	0.02
ALT (U/L)	26.0 ± 8.2	47.7 ± 23.3	<0.001
AST (U/L)	20.6 ± 3.9	72.4 ± 54	<0.001
WBC (10^9^/L)	5.9 ± 0.8	5.0 ± 3.8	0.23
Hgb (g/L)	132.2 ± 11.6	110.2 ± 24.0	<0.001
PLT (10^9^/L)	156.5 ± 23.7	85.9 ± 51.6	<0.001
ALP (U/L)	67.0 ± 14.0	160.0 ± 91.6	<0.001
PT (s)	N/A	14.3 ± 2.3	
Child-Pugh class (A/B/C)	N/A	27/2/1	
*Pyrosequecing data*			
Reads number	7225 ± 1033	7696 ± 922	0.08
Observed OTUs#	168 ± 28	172 ± 27	0.64
Chao1 index#	191 ± 29	198 ± 30	0.31
Shannon index#	3.63 ± 0.44	3.62 ± 0.43	0.93
Simpson index#	0.92 ± 0.06	0.93 ± 0.05	0.82

Abbreviation: BMI, body mass index; HBV, hepatitis B virus; PBC, primary biliary cirrhosis; Alb, albumin; TB, total bilirubin; ALT, alanine asninotrasferase; AST, aspartate aminotransferase; WBC, white blood cell; Hgb, hemoglobin; PLT, platelet; ALP, alkaline phosphatase; PT, prothrombin time; OTU, operational taxonomic unit.

^#^Indicate the alpha diversity was calculated after the reads number of each sample were equalized. N/A indicate the data was not measured in controls.
